# Disruptive behaviors among nurses in Israel – association with listening, wellbeing and feeling as a victim: a cross-sectional study

**DOI:** 10.1186/s13584-019-0340-6

**Published:** 2019-11-04

**Authors:** Sigal Shafran Tikva, Avraham N. Kluger, Yulia Lerman

**Affiliations:** 1 Hadassah University medical Hospital, Jerusalem, Israel; 2 Jerusalem College of Technology, Jerusalem, Israel; 3 School of Business Administration, The Hebrew University of Jerusalem, 91905 Jerusalem, Israel

**Keywords:** Disruptive behavior, Feeling as a victim, Listening, Nurses, Wellbeing

## Abstract

**Objectives:**

To examine the association between listening and disruptive behaviors and the association between disruptive behavior and the wellbeing of the nurse. To test whether constructive and destructive listening has an incremental validity.

**Methods:**

A structured questionnaire survey that measured the (constructive & destructive) listening climate at work, exposure to disruptive behaviors, well-being and feeling as a victim. We presented this survey using the Qualtrics software.

**Results:**

Of the final sample of 567 respondents who reported that they were nurses, M_Age_ = 38.41, 67% indicated that they were exposed to some form of disruptive behavior. Experiencing listening in the ward was associated with low levels of exposure to disruptive behaviors; exposure to disruptive behaviors, in turn, predicted reduction in the nurses’ wellbeing; the reduction in wellbeing was especially pronounced among nurses who felt like a victim. Each of the facets of the listening measure—constructive listening and destructive listening—had incremental validity in predicting exposure to disruptive behaviors. Finally, the effect of exposure to disruptive behavior on wellbeing was curvilinear.

**Conclusions:**

Disruptive behavior is a major challenge to the workplace well-being for nurses. The victim mentality has an adverse impact on nurses. Preventive efforts aimed at reducing disruptive behaviors among nurses and decreasing their sense of victimization are crucial for the well-being of nurses.

## Introduction

Disruptive behavior which is expressed in many different forms is an undesirable conduct among colleagues in the workplace. Disruptive behavior is sometimes referred to as lateral violence, bullying, workplace incivility, lateral hostility, horizontal hostility, horizontal violence, interpersonal conflict and disruptive behavior [5, 13, 31, 48]. The construct of disruptive behavior, using one name or another, among nurses has been discussed for over a century [8]. We chose the term “disruptive behavior” which refers to the negative behaviors among peer nurses following the term used by The Joint Commission [18] .

Disruptive behavior is commonly experienced by nurses around the world across cultures and borders [1, 2, 44], it also affects nursing students and new novice nurses [3, 9].

In Israel, a study aimed to describe the prevalence of ICU nurse bullying and what measures were taken to prevent it, showed that the levels of bullying were low to moderate (29%) and the level of prevention was weak or moderate. The higher the level of bullying, the lower the level of prevention [15].

Disruptive behavior has serious implications for the nurse, the organization and even the patient. With regard to the nurse, the literature reports physical and mental consequences that may result in weight loss, depression, sleep problems, anxiety, post-trauma-syndrome disorder (PTSD), and suicidal tendencies [5, 34, 37, 42]. For example, in a cohort study, bullying was found to be a predicting factor for mental health problems, such as anxiety, depression, and fatigue [36]. Exposure to disruptive behavior is also related to a decline in work satisfaction [35], increased burnout [1], and damaged relationships among colleagues [13].

However, disruptive behavior does not only harm the victimized nurse, it also has a negative impact both on the organization and the patients. In fact, disruptive behavior has a ripple effect because it leads to an increase in absenteeism, high turnover of nurses and tendency to leave the profession. A shortage of nurses could lead to damage in quality of care and decline in patients’ satisfaction [5, 13]. Indeed, bullying has a negative influence on nurse-assessed patient quality through their effect on perceptions of patient safety risk [42]. Moreover, nurses who are victims of disruptive behaviors tend to pay less attention to tasks, which increases the risk of making clinical errors [5], and adverse events [34]. Thus, it is not surprising that some reviews suggested that disruptive behavior “.. . can have a significant impact on care delivery, which can adversely affect patient safety and quality outcomes of care” [39], and that the Joint Commission (2008) stated, “Intimidating and disruptive behaviors can foster medical errors, contribute to poor patient satisfaction and to preventable adverse outcomes, increase the cost of care, and cause qualified clinicians, administrators and managers to seek new positions in more professional environments” [18].

Given the potential dire consequences of disruptive behavior, it is desirable to understand its antecedents. According to one review, the antecedents of workplace bullying fall into four main categories: role characteristics, quality of the relationship, leadership style and organizational culture [46]. Findings in another study showed three organizational factors that contribute to bullying and, the relationship between bullying and the resultant consequences: informal organizational alliances, organizational tolerance and reward of bullying and misuse of legitimate organizational processes and procedures [19].

In a study aimed to examine work climate, bullying and job performance, results showed that work bullying had a mediational role between most of the work climate dimensions and nurse outcomes [33]. Here, we focus on the role of quality of relationship. One key antecedent to relationship is listening quality. Indeed, current definitions of the construct of listening emphasize that relationship is one out of three components of the listening construct: attention, comprehension, and (positive) intention [20, 21]. Specifically, speakers develop a perception that they are being listened to when they perceive that the other person pays attention to them, understands them, and relates to them in a positive manner (non-judgmental, empathic, etc.). Moreover, empirical studies suggest that listening improves liking and relationships in all spheres of life, such as among strangers [29], and in marriage [6]. Moreover, employees who perceive that their supervisors listen, enjoy higher levels of job satisfaction [14, 45] and higher levels of psychological safety [7]. Similarly, listening was highly correlated with trust in dyads such as patient/physician [40], customer/salesperson [12], and suspect/detective [4]. We propose that listening among peers, such as among nurses, is also very likely to send signals of positive relationships, and thus be associated with a reduction in experiencing disruptive behaviors. To the best of our knowledge, the relationship between listening and the degree of exposure to disruptive behavior has not yet been investigated.

However, listening is correlated with lower levels of violence in domains other than nursing. Specifically, families, couples, marital, elderly people and children [11, 17, 23, 30, 32].

### Listening

Listening is a multi-dimensional construct that includes attention to the speaker, comprehension of the speaker, and a relational aspect, such as being empathic and non-judgmental [38]. Yet, measurement of perceived listening indicates that people tend to perceive “constructive” and “destructive” aspects of listening [25]. Therefore, in the current study, we sampled items tapping both the constructive and destructive aspects of listening.

#### Interventions

In a systematic review aimed to identify best practices for preventing and managing disruptive behaviors among staff nurses, the best method that was found to control and stop the phenomenon involves cognitive rehearsal of responses to common behaviors [27, 43]. Stagg et al., and Laschinger et al., found that authentic leadership had a negative direct effect on workplace bullying, which in turn had a direct positive effect on emotional exhaustion [27]. In a study aimed to evaluate role-play of bullying in nursing practice simulation as an active learning strategy, the results showed that role-play is a highly effective pedagogy, eliciting learning at both the cognitive and affective domains [47].

Several empirical studies of listening perceptions indicated that items reflecting good listening load on a separate factor than items reflecting poor listening. This led Kluger and Bouskila-Yam to propose the constructs of constructive listening and destructive listening [25] An example of an item showing high loading on constructive listening is “X tries hard to understand what I am saying”, and for destructive listening is “X discounts or explains away my feelings.” Indeed, Kluger and Zaidel [26] have shown not only that listening items form constructive and destructive listening factors, but they have differential validity [26]. Moreover, in a study of layperson theories of good listening that generated more than 70 listening items, items that were indicators of poor listening, did not load on the factor of good listening [28].

### Wellbeing

Well-being is good or satisfactory condition of existence; a state characterized by health, happiness, and prosperity. The symptoms of poor wellbeing are insomnia, poor mood, depression, decrease in motivation, self- evaluation etc.

Wellbeing is the balance point between an individual’s resource pool and the challenges faced [10].

In summary, in this study we tested the following model regarding disruptive behaviors:

### The objectives of the study

- To examine the association between listening and disruptive behaviors.
- To examine whether constructive and destructive listening has an incremental validity.
- To test the association between disruptive behavior and the wellbeing of the nurse.
- To test the role of feeling as a victim in augmenting the effects of disruptive behaviors on wellbeing.

## Methods

### Data source and participants

We developed a structured questionnaire survey to measure the (constructive & destructive) listening climate at work, exposure to disruptive behaviors, well-being and feeling as a victim. We conducted this survey using the Qualtrics software. First, we attempted to obtain permission to distribute the questionnaire from several managers in health care organizations, but we were refused. Thus, we distributed it via social network (Facebook), e-mails and the snowball method. We invited staff nurses to fill in the questionnaire: “Staff nurses, please access the questionnaire that deals with our behaviors, between each other, in daily work,. Filling out the questionnaire on Facebook and e-mails allows anonymous expression. The questionnaire is friendly, short and can be filled with a smartphone. We would appreciate your time.” In this way, we invited respondents to answer privately, without fear of supervisor involvement. This method also addressed concerns that respondents may have when answering questions regarding disruptive behavior. This distribution method allowed us to reach out to nurses from different organizations. Our invitation indicated that we were interested in examining behaviors between nurses; however, we did not mention “disruptive behaviors”, to avoid bias. The data collection lasted 2 months and we posted three reminders.

Prior to the distribution of this survey, we obtained an approval from the Institutional Review Board of the Jerusalem College of Technology.

### Measurements

Unless otherwise noted, we presented all items using a Likert scale ranging from 1 = *Does not reflect at all* to 7 = *Reflects to a very large degree*.

#### Disruptive behaviors

The main dependent variable was whether nurses had experienced disruptive behaviors from their nurses’ colleagues within the last 6 months prior to the study. Specifically, we asked “In the past six months, if and to which extent were you exposed to behaviors (listed below) from the colleague’s nurses in your workplace?” The disruptive-behavior list included experiencing negative remarks, verbal insults, humiliation in front of patients/staff member/ family, damaging authority, declining to assist with no reason, arrogant attitude, blaming, gossip and talking behind back, social isolation, and sexual harassment. A factor analysis indicated the presence of a single factor (only one factor had an eigenvalue > 1). A scale constructed from these items was reliable, α = .93.

#### Listening

We selected 12 items from the Facilitation Listening Survey [25] and adapted them to nurses. The items measured both constructive listening (seven items) and destructive listening (five items), however, we mixed the presentation of constructive and destructive items. Specifically, we asked “When nurses in my unit listen to each other or to me, most of the time they …” Examples of constructive-listening items are “Listen attentively”, “Allows another to fully express himself”, “Trying to understand what has been said”, and “Respects opinions even if they differ from theirs.” Examples of destructive-listening items are “Not interested in listening to others”, “Do not pay attention to what is said to them”, and “Speak back aggressively.” Both the constructive listening scale, α = .93, and the destructive-listening scale, α = .88, were reliable.

#### Wellbeing (symptoms of poor wellbeing)

We used 14 items to assess the wellbeing of the nurses by asking them for a rating of the degree to which they feel “Poor mood”, “Anxiety”, “Depression”, “Concentration difficulties”, “Insomnia”, “Changes in eating habits”, “Various types of pain”, “Absenteeism”, “Diminished quality of life off work”, “Decrease in self-evaluation”, “Decrease in motivation”, “Decrease in satisfaction”, “Turnover thoughts off the disruptive workplace”, and “Other”. Most respondents did not answer “Other”, so we discarded this item. Although a factor analysis indicated that these 13 items form three factors, they were highly correlated, and thus we created a single scale, α = .92. However, we also created sub-scales based on the factor analysis and labeled these scales Physiological Symptoms (e.g., “Insomnia”), α = .87, Motivation (e.g., “Decrease in motivation”), α = .90, and Negative Affect (e.g., “Depression”), α = .84.

#### Feeling as a victim

Out of the 10 items we developed to assess attitudes towards disruptive behaviors, a factor analysis indicated that four of these form a factor tapping victimhood. Because we deemed victimhood as a key outcome, we retained only these items. The items were “If I get hurt, I will quit”, “I ask for shifts without that person”, “When I see someone hurt, I know my *turn will arrive”, and “I feel as a victim”, α = .67.*

#### Socio-demographic

We also collected data regarding nurse’s age, gender, marital status, religion, type of organization working in (general hospital, geriatric, rehabilitation, etc.), work unit (ICU, internal medicine, etc.), and organization ownership (public, private or combined).

### Statistical analysis

We tested predictions about simple associations with Pearson correlations, and predictions regarding incremental validity and interaction with hierarchical-multiple regression.

## Results

A total of 637 respondents clicked the web link of the questionnaire. Yet, there were empty records or records with extensive missing data. We excluded these records and obtained a final sample of 567 respondents who reported that they were nurses (i.e., licensed, registered, or practical nurses), M_Age_ = 38.41, *SD* = 10.5, 90.3% female. Socio-demographic characteristics of this sample are presented in Table 1.

**Table 1:** Socio-demographic data of participants (*N* = 567)

Variable	*N*	%
*Gender*		
Female	511	90.3
Male	55	9.7
*Religiosity*		
Secular	378	76.8
Religious	63	12.8
Traditional	46	9.3
Ultra-Orthodox	5	1
*Educational level*		
Licensed practical nurse (LPN)	7	1.2
Registered nurse (RN)	83	14.7
Academic RN w BA	309	54.8
Academic RN w MA/Ph.D\	165	29.3
*Job rate*		
Full time	351	61.9
Part time	216	38.1
*Shifts*		
Mixed	300	53
Morning	197	34.8
Evening	37	6.5
Night	32	5.7
*Seniority in the current unit*		
Up to a year	79	14
Between 1 and 2 years	65	11.5
Between 2 and 4 Years	91	16.1
More than 4 years	331	58.5
*Organization*		
General hospital	370	75.4
Community care	75	15.3
Geriatric medical center	22	4.5
Psychiatric hospital	17	3.5
Rehabilitation	7	1.4
*Current unit*		
ICU	68	14
Internal	57	11.7
Surgery	52	10.7
Delivery & Obstetric & delivery room	49	10.1
Children	43	8.8
Operation Room	41	8.4
Emergency Room	32	6.6
Psychiatry	16	3.3
Ambulatory	11	2.3
Oncology	10	2.1
Other	107	22
*Ownership*		
Public	365	74
Private	45	9.1
Mixed	83	16.8

### Statistical analysis

**Fig. 1:**
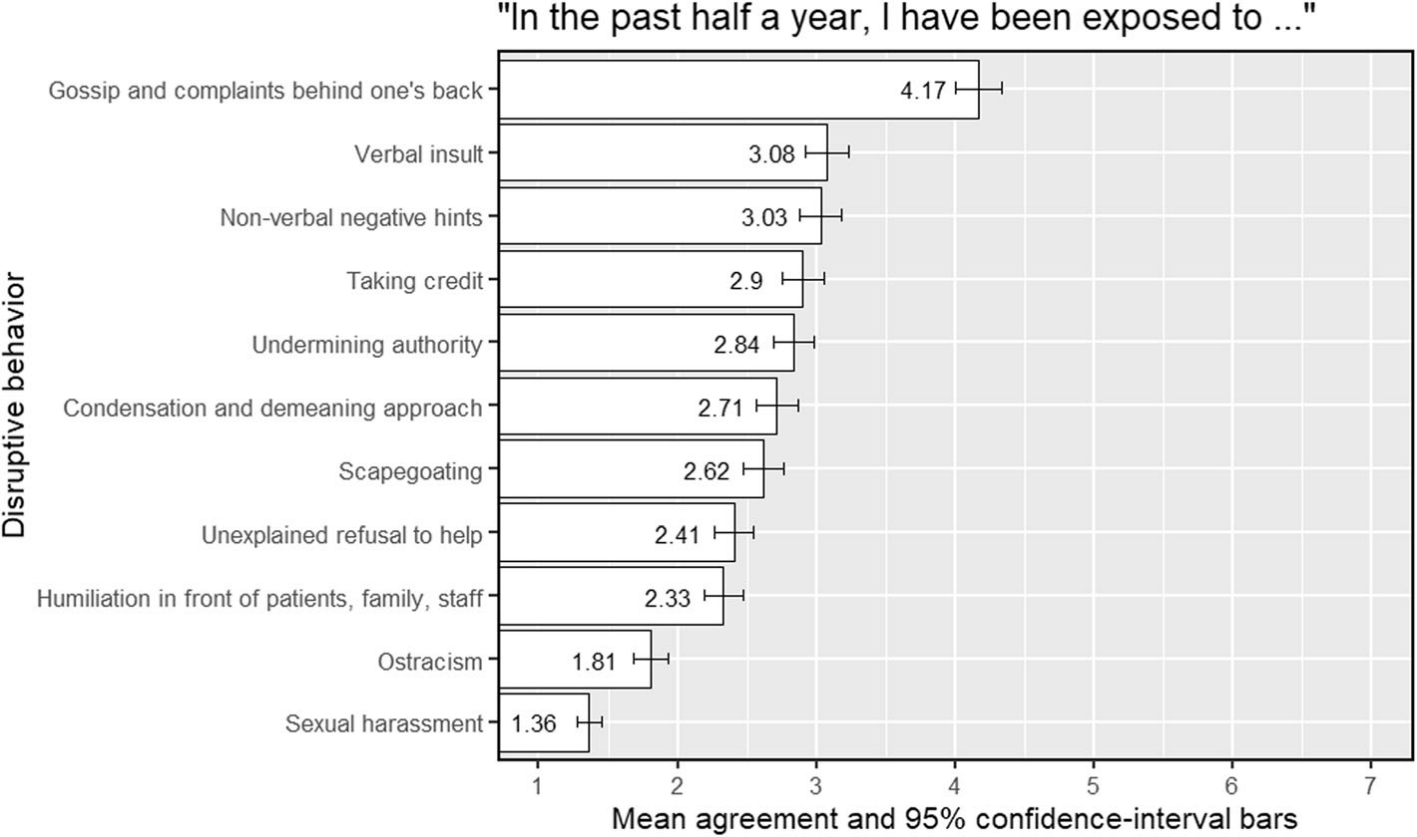
Mean of the exposure for each type of disruptive behavior

First, we probed the prevalence of all disruptive behavior and found that 67.2% of the respondents indicated that they were exposed to some form of disruptive behavior (those that were not exposed, marked 1 or other very low score on the rating scale of disruptive behaviors). Specifically, we present in Fig. 1 the mean of the exposure scales for each type of disruptive behavior in a descending order.

**Fig. 2:**
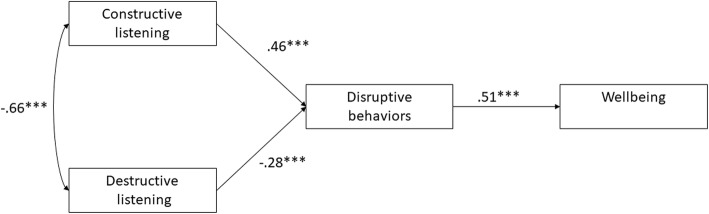
A path analysis demonstrating (**a**) incremental validity of two listening scales in predicting disruptive behaviors and (**b**) the role of disruptive behaviors as a mediator of the effects of listening on wellbeing

Second, we tested the correlations of the two listening scales with the disruptive-behavior scale and the remaining variables in our study. As can be seen in Table 2, the constructive- (destructive-) listening scale is negatively (positively) correlated with reporting exposure to disruptive behaviors. Third, we inspected the correlations between the listening scales, wellbeing, and subscales of wellbeing. As can be seen in Table 2, all of these correlations are significant, moderate in magnitude, and in the predicted direction. In addition, feeling as a victim showed the same pattern of correlations with the listening scales. To test the path model we used structural equation modeling (e.g., [24]). The results of this model are presented in Fig. 2. The model had a good fit to the data, χ^2^(2) = 4.40, *p* = .11, *RMSEA = .*05 [.00, .12], *SRMR* = .03. All the paths were significant at the .001 level.

**Table 2:** Means, standard deviations, and correlations

Variable	*M*	*SD*	1	2	3	4	5	6	7	8	9
Listening											
1. Constructive	5.03	1.19									
2. Destructive	2.70	1.3	−.66**								
3. Disruptive behavior	2.65	1.35	−.58**	.65**							
Wellbeing	2.89	1.39	−.35**	.39**	.51**						
5. Physical	2.80	1.38	−.25**	.30**	.40**	.93**					
6. Motivation	3.04	1.78	−.38**	.41**	.51**	.89**	.70**				
7. Negative Affect	2.05	1.02	−.28**	.32**	.43**	.85**	.72**	.71**			
8. Victim	2.58	1.33	−.23**	.30**	.39**	.50**	.43**	.48**	.43**		
9. Age	38.4	10.5	−0.01	0.07	−0.06	−.16**	−.15**	−.09*	−.15**	−0.07	
10. Gender	0.90	0.30	0.02	−.08*	−0.01	−0.03	−0.02	−0.04	−0.02	0.00	0.06

*Note.* * indicates *p* < .05; ** indicates *p* < .01. *M* and *SD* are used to represent mean and standard deviation, respectively

Notably, the strong correlation between destructive listening and disruptive behaviors, *r = −.66,* could be an artifact of shared content between these two scales. Specifically, the destructive-listening scale contains items such as “Speak back aggressively.” Such items are very similar to those of the disruptive behavior scale such as “condensation and demoing approach.” On the other hand, the constructive-listening scale does not contain any items resembling disruptive behaviors. Yet Fig. 2 shows that the constructive-listening scale has an incremental validity in predicting disruptive behavior, such that item overlap between constructs could explain some, but definitely not all of the association between listening and the experience of disruptive behavior.

**Fig. 3:**
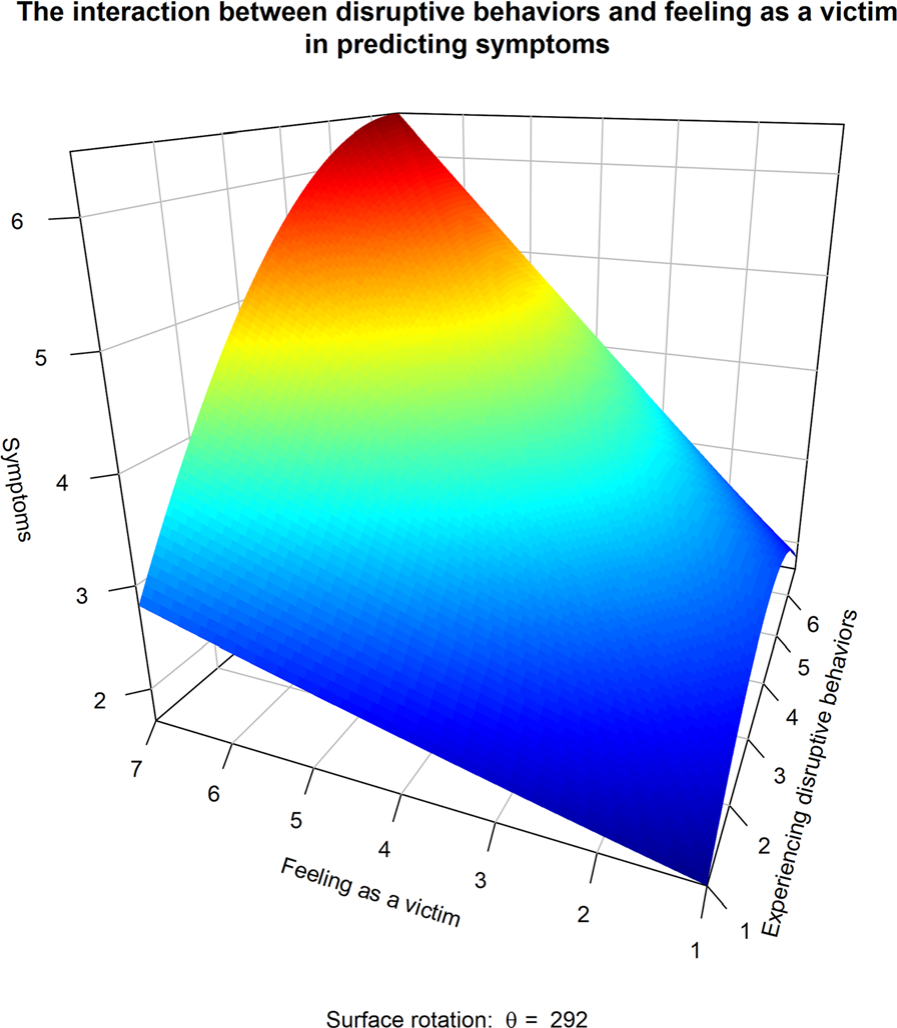
Response surface plot

Finally, we tested the interaction between experiencing disruptive behavior and feeling as a victim in predicting wellbeing. Typically, interactions were tested with the following model Y = a + b_1_X_1_ + b_2_X_2_ + b_3_X_1_X_2_, where significance of b3 indicates the presence of an interaction. However, a stringiest test [16] needs to allow for the possibility of non-linear effects of either X_1_, X_2_, or both, as follows Y = a + b_1_X_1_ + b_2_X_2_ + b_3_X1^2^ + b_4_X2^2^ + b_5_X_1_X_2_, where the significance of b5 indicates the presence of an interaction, controlling both for the main effects of the predictors and for their curvilinear effects. The results of this test are presented in Table 3 and Fig. 3.

**Table 3:** Predicting wellbeing from exposure to disruptive behavior, feeling as a victim, non-linear effects of the above, and an interaction between them

Predictor	β	*t*	*P*
Disruptive	.45	9.67	< .0001
Disruptive^2^	−.15	−3.85	< .0002
Feeling as victim	.34	7.23	< .0001
Feeling as victim^2^	.00	0.13	=.90
Disruptive * Feeling as victim	.13	3.66	< .0002

Given that age was negatively correlated with wellbeing and symptoms (see Table 2), we tested whether controlling for age would alter any of our conclusions. It did not. Specifically, adding age as a predictor of wellbeing in our path model (Fig. 2), changed the standardized path from disruptive behavior to wellbeing from .51 (Fig. 2) to .49. Similarly, controlling for age in the polynomial regression in Table 3 changed the standardized coefficient of the interaction term from .132 (Table 3) to .124, t = 3.40, *p* = .0007. Thus, age cannot serve as an alternative explanatory variable to our results.

## Discussion

The results of a cross-sectional study of 567 Israeli nurses largely support our model. Experiencing listening in the ward was associated with experiencing low levels of exposure to disruptive behaviors; exposure to disruptive behaviors, in turn, predicted a reduction in the nurses’ wellbeing; however, the reduction in wellbeing was especially pronounced among nurses who felt like a victim. In addition, we have shown that each of the facets of the listening measure—constructive listening and destructive listening—had an incremental validity in predicting exposure to disruptive behaviors. Finally, we found, although we did not anticipate it, that the effect of exposure to disruptive behavior on wellbeing was curvilinear. Specifically, at a low range of exposure, there were no apparent effects on wellbeing, but at a high level of exposure, every increment in exposure translated into an accelerating damage to wellbeing, where this effect was especially pronounced among nurses who felt as a victim.

Our findings about the importance of listening in buffering exposure to disruptive behaviors joins other findings suggesting that listening is associated with low levels of violence [11, 17, 23, 30, 32]. It may hint that training staff in listening to each other may contribute to a reduction in disruptive behavior. One way to change quickly the listening behavior among nurses could involve “Listening Circles”, also known as “The Council” [21]. Indeed, participating in “Listening Circles” at the workplace have been shown to reduce attitude extremity [21], which may hint that not only such training can increase listening, but that it may also reduce disruptive behaviors.

Training nurses in listening, which is an important communication skill, can have benefits beyond reducing disruptive behaviors. Specifically, nurses who listen well may contribute to the wellbeing of their patients [41]. Indeed, deliberate listening by nurses seems to reduce depression among mothers to premature newborns; in addition, listening by the medical staff, including nurses, is associated with satisfaction with hospitalization [22], and reducing worries of mothers to newborns [49].

Second, our findings replicate previous ones showing that exposure to disruptive behavior is associated with reduced wellbeing. These results point out the irony that the medical staff who are supposed to heal the sick, nevertheless often behave in a way that has a negative effect on other nurses and consequently on patients. Third, our work suggests that exposure to disruptive behavior does not necessarily lead to a reduction in wellbeing. Specifically, it appears that only some people, who are exposed to high levels of disruptive behaviors, are at risk of reduced wellbeing. Nurses who feel as a victim are especially prone to the damage of exposure to disruptive behaviors. Thus, it might be desirable to identify nurses that are at risk and to consider interventions geared toward reducing the feeling as a victim. Such as support groups, facilitating personal sessions with a social worker or psychologist from the organization, etc.

### Generalization

We recruited nurses into our study through social media and snowball sampling. This method has the advantage of sampling nurses across multiple medical establishments and specialties. This sampling increases our ability to generalize our findings to many types of nurses. However, our sample might be biased such that certain types of nurses chose to participate (like those who spend more time surfing the internet). In addition, our study was done in Israel. Yet, the components of our model were tested and validated in other cultures (e.g., the link between listening and low level of violence among non-nurses, the link between exposure to disruptive behaviors and wellbeing); thus, our results are likely to be generalizable to other cultures.

### Limitations

The most obvious limitation of our study was its cross-sectional design. Future research could attempt to replicate our findings with an experimental design. For example, wards could be randomized into participating, or placed on a waiting list, for listening training, and levels of disruptive behaviors before and after the training could be measured. Similarly, nurses who feel like a victim could be randomized into training design to reduce these feelings and changes in the wellbeing of those nurses could be tracked, especially among those who work in a ward characterized by high levels of disruptive behaviors. We believe that our results that are based on a relatively large sample justify the extra effort needed to re-test our model with experimental design in the field.

## Conclusions

Past research indicates that exposure to disruptive behaviors among nurses is negatively associated with their wellbeing. We replicated these results and offered a model extending these findings to explain both one antecedent of disruptive behavior (listening) and one moderator (feeling as a victim). Specifically, we have shown that nurses who perceive their colleagues to be highly adept at listening also report low levels of exposure to disruptive behaviors. This may suggest that training nurses in listening skills could contribute to their wellbeing by reducing the incidents of disruptive behaviors. In addition, we have shown that the negative effects on wellbeing emanating from exposure to disruptive behavior are especially acute among nurses who felt as a victim. “Interventions targeted to this specific group could help them cope better with disruptive behavior.”

In sum, our findings suggest that good quality listening skills among medical staff members will improve the quality of the work life of the nurses, and consequently contribute to the wellbeing of all people interacting with them.

## Data Availability

The datasets used and/or analysed during the current study available from the corresponding author on reasonable request.
